# Beyond Tumor Control in Sporadic Neuroendocrine Tumor: Metabolic and Bone Health During Treatment with Somatostatin Analogs

**DOI:** 10.3390/jcm15176927

**Published:** 2026-09-07

**Authors:** Roberta Modica, Alessia Liccardi, Roberto Minotta, Elio Benevento, Gianfranco Di Iasi, Massimo Di Nola, Michele Coletta, Colao Annamaria

**Affiliations:** Endocrinology, Diabetology and Andrology Unit, Department of Clinical Medicine and Surgery, University of Naples Federico II, 80131 Naples, Italy; robertominotta@gmail.com (R.M.); elio.benevento@gmail.com (E.B.); gianfrancodiiasi@gmail.com (G.D.I.); massimodinola96@gmail.com (M.D.N.); michele99coletta@gmail.com (M.C.); colao@unina.it (C.A.)

**Keywords:** neuroendocrine, neuroendocrine tumor, somatostatin analog, bone, osteoporosis, osteopenia, glycemic control, calcium metabolism

## Abstract

**Background**: Somatostatin analogs (SSAs) are widely used in the treatment of neuroendocrine tumors (NETs). Despite SSAs having a favorable safety profile, their long-term impact on glucose metabolism and bone health remains uncertain. This study aimed to compare glycemic, calcium-phosphorus, and densitometric parameters between patients with sporadic NETs receiving SSA therapy and untreated patients. **Methods**: A retrospective observational study enrolling patients with sporadic NETs was conducted at Federico II University of Naples, Endocrinology Unit, ENETS Center of Excellence. **Results**: A total of 40 patients were enrolled: 18 (45%) treated with SSA (SSA+) and 22 (55%) untreated (SSA−). The median duration of SSA treatment was 24.5 months (range 12–180). HbA1c was significantly higher in the SSA+ group compared to SSA− (median 6.20% vs. 5.70%; *p* = 0.027). Corrected calcium levels were significantly lower in SSA+ patients (8.90 vs. 9.47 mg/dL; *p* = 0.045). No significant differences were observed between groups in phosphorus, vitamin D or PTH levels. Median lumbar T-score was −0.15 in SSA+ vs. −1.10 in SSA− (*p* = 0.256); lumbar BMD was 1.23 vs. 0.97 g/cm^2^ (*p* = 0.127). Femoral neck T-score was −0.85 vs. −1.55 and femoral BMD was 0.89 vs. 0.78 g/cm^2^. The prevalence of osteoporosis and osteopenia did not differ significantly between groups. Within the SSA+ group, treatment duration showed no significant correlations with any densitometric parameter. **Conclusions**: SSA treatment in patients with sporadic NETs is associated with a significant impairment of glycemic control. The reduction in serum calcium observed in SSA+ patients may indicate an indirect effect on calcium-phosphorus metabolism that warrants further investigation. These findings support careful metabolic assessment during follow-up. Although routine densitometric evaluation based solely on SSA exposure does not appear warranted, bone health assessment may be considered according to individual skeletal risk factors and the patient’s overall clinical profile.

## Simple Summary

Neuroendocrine tumors are uncommon tumors that frequently require prolonged treatment with somatostatin analogs, to control tumor growth and hormone-related symptoms. Although these agents have a generally favorable safety profile, their long-term effects on glucose metabolism and skeletal health remain incompletely understood. This study compared metabolic and bone parameters in patients with neuroendocrine tumors receiving somatostatin analogue therapy and in untreated patients, including glycemic control, calcium balance, and bone mineral density. Treated patients showed higher blood glucose levels and lower calcium levels than untreated patients, whereas bone density did not differ significantly between the two groups. Notably, a substantial proportion of patients in both groups presented reduced bone density. Given the observational design and relatively limited sample size, these findings indicate associations rather than causal relationships. These findings highlight the importance of considering metabolic and skeletal health during the clinical follow-up of patients with neuroendocrine tumors, with bone assessment tailored to individual risk factors.

## 1. Introduction

Neuroendocrine neoplasms (NEN) are rare tumors, with an estimated annual incidence of 3–5 cases per 100,000 individuals [1]. They originate from neuroendocrine cells, widely distributed throughout the body and capable of producing and secreting a variety of bioactive amines and peptides. Although functioning NEN can cause distinct clinical syndromes, the majority of these tumors are non-functioning [2,3]. The gastrointestinal tract is the most common site of NEN (67%) followed by thoracic NEN [4,5].

NETs generally follow an indolent course, although they are often locally advanced or metastatic at the time of diagnosis, which typically necessitates long-term, sometimes lifelong, therapy. In contrast, neuroendocrine carcinomas exhibit more aggressive behavior and predominantly require chemotherapy [6]. Managing these tumors remains challenging for clinicians; however, the range of therapeutic options has significantly expanded over recent decades. Surgery continues to be the primary treatment with curative intent for resectable disease, and it may also serve a palliative role in advanced cases. Systemic therapies for NEN are selected according to on tumor grade, disease burden, growth rate, and patient performance status and can be administered as first-line treatment, in an adjuvant setting following surgery, or for palliation. Currently, the main available systemic options include somatostatin analogs (SSAs), molecular targeted therapies such as everolimus and sunitinib, peptide receptor radionuclide therapy (PRRT), and chemotherapy [6].

The prolonged survival of patients with NETs, even in the metastatic setting, may at least partially explain the increased risk of cardiovascular events, gastrointestinal disorders, osteoporosis, and sarcopenia, as demonstrated in retrospective studies [7]. Moreover, both the tumor and systemic therapies can adversely affect nutritional status, leading to deficiencies in several vitamins, particularly vitamin D, which negatively impacts bone metabolism [8,9,10]. Patients treated with SSAs showed significantly lower vitamin D and calcium levels, mainly caused by iatrogenic malabsorption [11,12]. The impact of NEN on bone health is also influenced by the occurrence of bone metastases which, although less common than hepatic and lymph-node involvement, represent a frequent metastatic site. When present, bone metastases contribute to a substantial skeletal burden, including pathological fractures, bone pain, spinal cord compression, and, in some cases, hypercalcemia, ultimately leading to impaired bone integrity, reduced mobility, and a significant deterioration in quality of life [13,14,15]. In patients with NEN, bone metastases are observed more frequently in males and in patients with a pulmonary primary [13]. Their presence is associated with worse clinical outcomes and is considered an adverse prognostic factor. Evidence from large European and US registries, as well as multicenter observational studies, consistently confirms that skeletal involvement correlates with reduced overall survival, increased skeletal-related events, and a greater burden of morbidity, emphasizing the significant clinical impact of bone metastases in this population [16,17,18,19,20].

**Figure 1:**
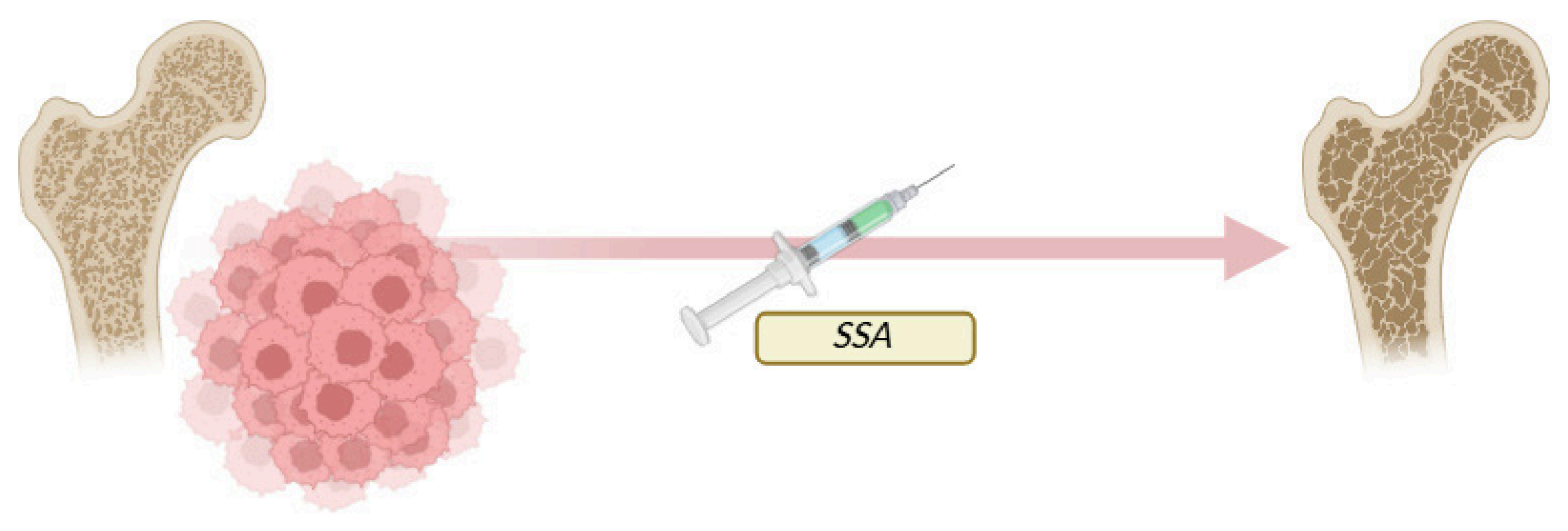
Schematic overview of the potential metabolic and skeletal implications of somatostatin analog therapy in patients with neuroendocrine tumors. The figure illustrates the possible association of SSA treatment with alterations in glucose and calcium metabolism and their potential relationship with bone health. The diagram is conceptual and does not imply a causal relationship.

This study aimed to compare glycemic parameters, calcium-phosphorus metabolism, and bone health between patients with sporadic NETs receiving SSA therapy and untreated patients, with particular attention to osteopenia and osteoporosis. Figure 1 provides a schematic overview of the potential metabolic and skeletal implications of SSA therapy investigated in the present study, including alterations in glucose and calcium metabolism and their possible relationship with bone health.

## 2. Materials and Methods

Data from patients with sporadic NETs referred to the Endocrinology Unit of the ENETS Center of Excellence at the University of Naples “Federico II” between 2015 and 2025 were retrospectively analyzed. Although this study included patients treated between 2015 and 2025, the data collection and analysis for the present study were initiated only after obtaining ethical approval and authorization. Eligible participants met the following inclusion criteria: diagnosis of sporadic NETs and treatment with SSA for at least 12 months, with a dual-energy X-ray absorptiometry (DEXA) scan available during the observation period. SSA-treated patients received either lanreotide Autogel at the standard dose of 120 mg every 28 days or octreotide LAR at the standard dose of 30 mg every 28 days. No dose escalation or treatment modification occurred during the observation period. The untreated control group (SSA−) comprised patients with sporadic NETs not receiving SSA therapy during the same period. Patients with bone metastases or those receiving antiresorptive therapy were excluded. Metabolic parameters including HbA1c, fasting glucose, corrected calcium calculated using the following formula: corrected calcium (mg/dL) = measured total calcium (mg/dL) + 0.8 × [4.0 − serum albumin (g/dL)], phosphorus, 25-OH vitamin D, and parathyroid hormone (PTH) were collected from clinical records at the time closest to the DEXA scan. HbA1c measurements were available for all patients included in the study. Bone mineral density (BMD), T-scores, Z-scores, and site-specific measurements at the lumbar spine and femoral neck were collected from all available DEXA scans in both groups. DEXA examinations were not performed using the same scanner or interpreted by the same operator; however, all measurements were obtained according to standardized DEXA acquisition and interpretation procedures. BMD measurements obtained by DEXA were used to classify patients according to the World Health Organization (WHO) criteria. Osteopenia was defined as a T-score between −1.0 and −2.5 standard deviations (SD) below the mean for a young adult reference population, while osteoporosis was defined as a T-score of −2.5 SD or lower. T-scores, Z-scores, and site-specific BMD values at the lumbar spine and femoral neck were recorded for all patients.

Descriptive statistics were generated for the study population. Fisher’s exact test for categorical variables and the Mann–Whitney U test for continuous variables were used for the statistical analysis. All analyses were performed using SPSS Statistics software, version 23.0 (IBM Corporation, New York, NY, USA).

The study protocol was approved by the Ethics Committee of the University of Naples “Federico II” (approval no. 259/2023), and written informed consent was obtained from all participants.

## 3. Results

A total of 40 patients with sporadic NETs were enrolled: 18 (45%) treated with SSA (SSA+) and 22 (55%) untreated (SSA−). The two groups were comparable in terms of age (median 64 years in both groups; *p* = 0.481), BMI (28.1 vs. 25.5 kg/m^2^; *p* = 0.286), and sex distribution (61% vs. 77% female; *p* = 0.315). The median duration of SSA treatment was 24.5 months (range 12–180 months). Among the 18 SSA-treated patients, 10 (55.6%) received lanreotide Autogel and eight (44.4%) received octreotide LAR. All patients received the standard approved dose, with no dose escalation or treatment modification during the observation period. Diabetes mellitus was present in five of 18 patients (27.8%) in the SSA+ group and in seven of 22 patients (31.8%) in the SSA− group.

Regarding metabolic parameters, HbA1c was significantly higher in the SSA+ group compared to SSA− (median 6.20% vs. 5.70%; *p* = 0.027). Corrected calcium levels were significantly lower in SSA+ patients (8.90 vs. 9.47 mg/dL; *p* = 0.045). No significant differences were observed between groups in phosphorus (*p* = 0.342), vitamin D (*p* = 0.449), or PTH levels (*p* = 0.653).

**Figure 2:**
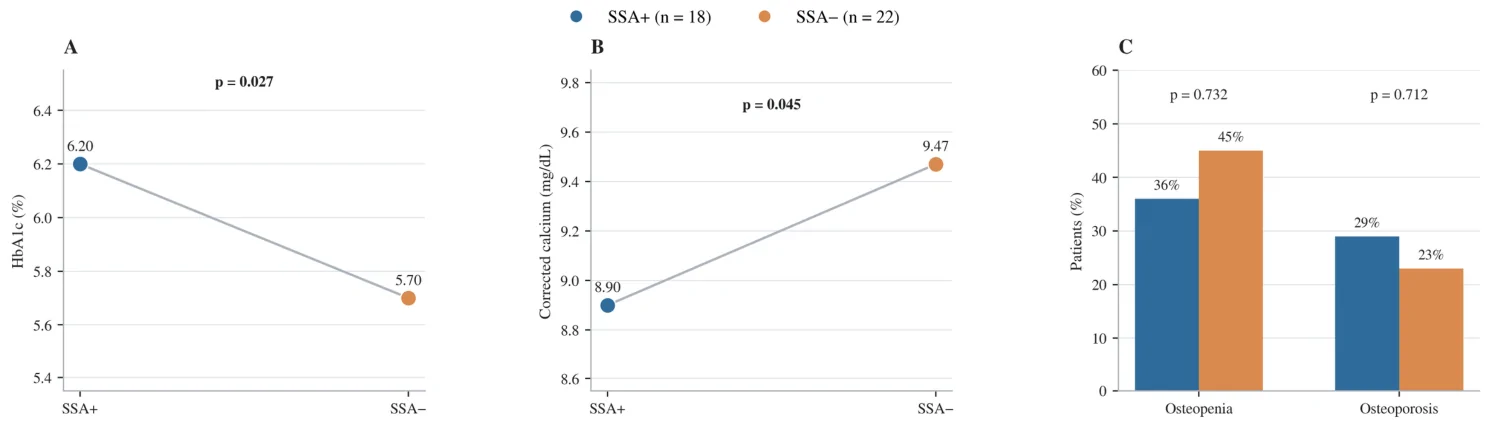
Metabolic and bone health assessment in patients receiving SSA therapy (SSA+) and patients not receiving SSAs (SSA−). (**A**) Median HbA1c levels. (**B**) Median corrected calcium levels. (**C**) Prevalence of osteopenia and osteoporosis according to SSA treatment status.

Regarding bone density, median lumbar T-score was −0.15 in SSA+ vs. −1.10 in SSA− (*p* = 0.256); lumbar BMD was 1.23 vs. 0.97 g/cm^2^ (*p* = 0.127). Femoral neck T-score was −0.85 vs. −1.55 (*p* = 0.570) and femoral neck BMD was 0.89 vs. 0.78 g/cm^2^ (*p* = 0.307). The prevalence of osteoporosis (29% vs. 23%; *p* = 0.712) and osteopenia (36% vs. 45%; *p* = 0.732) did not differ significantly between groups. Overall, the majority of the whole cohort (65%) exhibited impaired bone health (osteopenia or osteoporosis). Within the SSA+ group, treatment duration showed no significant correlations with any densitometric parameter (Table 1 and Figure 2).

## 4. Discussion

In this retrospective monocentric observational study of patients with sporadic NETs, SSA treatment was associated with higher HbA1c and lower corrected serum calcium levels compared with patients not receiving SSA treatment. No statistically significant differences were observed in BMD or in the prevalence of osteopenia and osteoporosis between the two groups. Nevertheless, a high overall prevalence of impaired bone health was detected in the entire cohort, with 65% of patients presenting with osteopenia or osteoporosis. These findings highlight the importance of metabolic assessment during follow-up. Bone health evaluation should be individualized according to conventional skeletal risk factors and the patient’s overall clinical profile.

Metabolic Effects of Somatostatin Analogs

The significantly higher HbA1c values observed in SSA-treated patients in our cohort are consistent with well-established evidence that SSAs impair glucose homeostasis [21,22]. This effect is mainly related to the inhibition of pancreatic insulin secretion, although the concomitant suppression of glucagon may result in a variable metabolic response among patients [23,24,25]. However, the observational design of our study does not allow a causal relationship between SSA treatment and higher HbA1c to be established.

Our findings are consistent with a retrospective cohort study of 279 patients with NETs, in which SSA therapy was associated with a mean increase in HbA1c of 3.30 ± 6.30 mmol/mol (*p* < 0.001) [26]. Among 209 evaluable patients, 19 developed type 2 diabetes, while five of 31 patients with pre-existing diabetes required intensification of antidiabetic therapy. Although these data support careful assessment of glucose metabolism during SSA therapy, prospective studies are needed to clarify the magnitude and clinical relevance of this association [26,27,28].

The findings from the aforementioned cohort and the present study indicate that worsening glycemic control may occur independently of BMI changes. In the previous cohort, BMI decreased by 1.04 ± 2.79 kg/m^2^ (*p* < 0.001). This apparent paradox, namely, the worsening of glucose metabolism in the context of weight loss, reflects the complex interplay between SSA-induced insulin suppression and the weight-reducing effects of reduced insulinemia, together with a potential contribution from SSA-induced exocrine pancreatic insufficiency and carbohydrate malabsorption [26,29,30,31]. In our cohort, the median HbA1c in SSA-treated patients was 6.20% versus 5.70% in untreated controls (*p* = 0.027), and while the groups were comparable in BMI (28.1 vs. 25.5 kg/m^2^; *p* = 0.286), this difference did not reach statistical significance, likely due to the relatively limited sample size.

The metabolic implications of SSA-induced dysglycemia extend beyond glycemic control per se. Diabetes mellitus is a well-recognized independent risk factor for skeletal fragility through multiple mechanisms including impaired bone quality, altered bone turnover, and increased fracture risk independent of BMD [15,32,33]. In the context of patients with NETs already predisposed to bone loss through disease-related and nutritional factors, the additive contribution of SSA-induced glucose impairment to skeletal vulnerability deserves explicit clinical attention. Metabolic management may also have broader clinical implications in patients with NETs. In the retrospective multicenter PRIME-NET study, metformin use was associated with longer progression-free survival in patients with diabetes and advanced pancreatic NETs receiving SSA or everolimus [34]. Although these findings do not establish a causal or mechanistic relationship, they support further investigation of the potential interplay between glucose metabolism, antidiabetic treatment, and clinical outcomes in patients with NETs. A post hoc analysis of the CLARINET trial further suggested that metformin use in patients with diabetes and NETs in the placebo arm was associated with longer progression-free survival (PFS) (85.7 vs. 38.7 weeks compared to patients not on metformin), whereas the effect was attenuated in patients receiving lanreotide, possibly because lanreotide itself already inhibits mTORC1 signaling [34]. These findings collectively argue for proactive glycemic management in SSA-treated patients with NETs, with early consideration of metformin where appropriate, pending prospective confirmation [35,36].

The absence of a significant difference in phosphorus, vitamin D, and PTH levels between the two groups in our cohort was notable. Regarding vitamin D, these results are consistent with available data showing no significant influence of SSA therapy on vitamin D concentrations in patients with NETs after adjusting for other variables [37]. However, the small sample size may have limited our ability to detect subtle differences, and the absence of systematic supplementation data represents a potential confounder. The relevance of vitamin D status in patients with NETs with bone involvement is underscored by a multicenter retrospective study of 291 patients with well-differentiated gastroenteropancreatic NET (GEP-NET), The study identified severe vitamin D deficiency (25(OH)vitamin D < 10 ng/mL) as independently associated with the presence of fragility fractures at diagnosis (odds ratio 5.9; 95% CI 1.2–27.8; *p* = 0.03) [38].

Somatostatin Analogs Effects on Bone Health

No statistically significant differences in BMD or in the prevalence of osteopenia and osteoporosis were observed between SSA-treated and untreated patients. Nevertheless, the overall burden of low bone mass in the cohort was substantial, with 65% of enrolled patients presenting with osteopenia or osteoporosis. This prevalence is consistent with the approximately threefold higher odds of osteopenia/osteoporosis reported in patients with GEP-NET compared with age-matched controls in a single-center case–control study (odds ratio 3.17; 95% CI 1.16–7.8; *p* < 0.001). The study included 90 patients with GEP-NET and 50 controls and reported significantly lower BMD at all skeletal sites in patients, even at an early disease stage [39]. These data, together with findings showing an approximately twofold higher risk of fragility fractures in patients with GEP-NET compared with the general population (odds ratio 2.0; 95% CI 1.1–3.6; *p* = 0.02), with the difference persisting even among patients in Stages I–II (17.1% vs. 8.2%; *p* < 0.01), confirm that skeletal vulnerability in patients with NETs is not merely a consequence of advanced or metastatic disease [38].

SSA-treated patients had numerically higher lumbar T-scores and BMD than untreated patients, but these differences were not statistically significant. These numerical differences should not be interpreted as evidence of a protective effect of SSAs on bone. Differences in clinical characteristics, follow-up, nutritional status, or vitamin D and calcium supplementation may have influenced these findings. However, these factors were not systematically assessed. The limited sample size may also have reduced the ability to detect clinically meaningful between-group differences. This interpretation is supported by longitudinal data showing that 82% of patients with GEP-NET with hypovitaminosis D received appropriate vitamin D supplementation, with a consequent significant increase in mean 25(OH)vitamin D from 22.6 ± 11.6 ng/mL at baseline to 30.1 ± 11.2 ng/mL at follow-up (*p* < 0.01) [38]. The significantly lower corrected calcium levels in SSA-treated patients (8.90 vs. 9.47 mg/dL; *p* = 0.045) observed in our study are consistent with previous evidence suggesting that SSA may affect intestinal and pancreatic secretions and contribute to nutrient malabsorption. The absence of a compensatory elevation of PTH in our SSA-treated patients despite lower calcium is noteworthy, and may reflect either adequate baseline PTH reserve or a subclinical degree of calcium depletion insufficient to trigger secondary hyperparathyroidism. By contrast, a significantly higher prevalence of secondary hyperparathyroidism (SHPT) due to vitamin D deficiency was reported in patients with GEP-NET with fractures compared with those without fractures (45% vs. 22%; *p* = 0.04), highlighting the clinical relevance of monitoring this metabolic axis in patients with NETs [38].

Bone health in patients with NETs is likely influenced by multiple factors, including age, sex, menopausal status, nutritional status, vitamin D deficiency, sarcopenia, tumor characteristics, and comorbidities [7,8,39,40,41,42]. Patients with bone metastases were excluded from the present study because metastatic skeletal disease represents a distinct cause of bone morbidity [13,14,43]. Accordingly, our findings apply only to patients without clinically identified bone metastases.

SSA treatment duration was not significantly correlated with any densitometric parameter. This finding is consistent with previous longitudinal evidence showing no significant association between SSA use and incident fractures [38,44]. However, the SSA-treated subgroup included only 18 patients, and bone turnover markers were not systematically available. Therefore, a clinically relevant association between treatment duration and skeletal outcomes cannot be excluded.

Disease-related factors may also contribute to impaired bone health in patients with NETs. Serum serotonin levels have been inversely associated with hip BMD, while alterations in the kynurenine pathway may also affect osteoblast activity [15,45]. Vitamin D deficiency and sarcopenia are common in this population and may further increase skeletal vulnerability. In a case–control study, patients with GEP-NET had lower skeletal muscle mass than controls, and muscle mass was positively correlated with lumbar spine and total hip BMD [39].

Bone metastases represent an additional and well-established contributor to skeletal morbidity in patients with NETs and are associated with poorer survival and a higher risk of skeletal-related events. In lung NETs specifically, women showed a lower prevalence of bone metastases compared to men, and hypovitaminosis D at diagnosis was independently associated with subsequent development of bone metastases [13]. Although metastatic bone disease was outside the scope of the present analysis, these findings emphasize that BMD assessment must be contextualized within a comprehensive skeletal health evaluation [46,47].

## 5. Study Limitations

This study has several limitations. First, its retrospective, single-center design and relatively small sample size, particularly in the SSA-treated subgroup (n = 18), limited the statistical power and may have prevented the detection of clinically meaningful differences in skeletal outcomes. Moreover, the observational design does not allow causal relationships to be established and remains susceptible to selection bias, information bias, and residual confounding. The biological and clinical heterogeneity of NETs represents a substantial limitation. Metabolic and skeletal outcomes may be influenced by several tumor-, treatment-, and patient-related factors, including primary tumor site, grade and disease stage, pancreatic involvement or previous pancreatic surgery, diabetes and antidiabetic treatment, renal function, menopausal status, smoking, and vitamin D or calcium supplementation. The limited sample size precluded a robust multivariable analysis adjusting for these covariates. Therefore, residual confounding cannot be excluded, and the observed associations should be considered exploratory and interpreted with caution. In addition, DEXA examinations were not uniformly performed using the same scanner or interpreted by the same operator, and potential inter-scanner and inter-operator variability cannot therefore be excluded. Similarly, bone turnover markers were not systematically available, and imaging was not uniformly performed to exclude subclinical skeletal disease. Collectively, these limitations prevent determination of the independent contribution of SSA therapy to the observed metabolic and skeletal findings. Larger prospective multicenter studies should include standardized longitudinal assessment of glycemic parameters, calcium-phosphorus metabolism, bone turnover markers, vitamin D and calcium supplementation, nutritional status, body composition, and BMD, with appropriate adjustment for relevant tumor-, treatment-, and patient-related confounders.

## 6. Conclusions

In this retrospective observational study, SSA treatment was associated with higher HbA1c and lower corrected calcium levels in patients with sporadic NETs, whereas no statistically significant differences were observed in BMD or in the prevalence of osteopenia and osteoporosis between SSA-treated and untreated patients. Nevertheless, the high overall prevalence of reduced bone mass suggests that skeletal health represents a clinically relevant aspect of the long-term management of patients with NETs, regardless of treatment status. These findings should be interpreted cautiously due to acknowledged study limitations. Evaluation of bone health should be individualized according to conventional risk factors and the patient’s overall clinical profile, rather than being recommended solely on the basis of SSA exposure. Prospective longitudinal studies are needed to clarify the potential skeletal effects of long-term SSA treatment.

## Figures and Tables

**Table 1:** Patient characteristics and metabolic/densitometric parameters.

Parameter	SSA+ (n = 18)	SSA− (n = 22)	*p*-Value
*Baseline characteristics*			
Age, years, median	64	64	0.481
BMI, kg/m^2^, median	28.1	25.5	0.286
Female sex, %	61	77	0.315
SSA treatment duration, months, median (range)	24.5 (12–180)	—	—
*Biochemical parameters*			
HbA1c, %, median	6.20	5.70	0.027
Corrected calcium, mg/dL, median	8.90	9.47	0.045
*Densitometric parameters (n = 40 evaluable)*			
Lumbar spine T-score, median	−0.15	−1.10	0.256
Lumbar spine BMD, g/cm^2^, median	1.23	0.97	0.127
Femoral neck T-score, median	−0.85	−1.55	0.570
Femoral neck BMD, g/cm^2^, median	0.89	0.78	0.307
Osteoporosis, %	29	23	0.712
Osteopenia, %	36	45	0.732

SSA: somatostatin analog; BMD: bone mineral density.

## Data Availability

The data presented in this study are available on request from the corresponding authors.

## Abbreviations

The following abbreviations are used in this manuscript:

BMD	bone mineral density
DEXA	dual-energy X-ray absorptiometry
GEP-NET	gastroenteropancreatic NET
NEN	neuroendocrine neoplasms
NET	neuroendocrine tumors
PFS	progression-free survival
PRRT	peptide receptor radionuclide therapy
PTH	parathyroid hormone
SD	standard deviations
SHPT	secondary hyperparathyroidism
SSA	somatostatin analogs
WHO	World Health Organization

## References

[1] Modica R., Liccardi A., Minotta R., Cannavale G., Benevento E., Colao A. (2024). Current Understanding of Pathogenetic Mechanisms in Neuroendocrine Neoplasms. Expert Rev. Endocrinol. Metab..

[2] Spada F., Rossi R.E., Modica R., Gelsomino F., Rinzivillo M., Rubino M., Pisa E., La Salvia A., Fazio N. (2025). Functioning Neuroendocrine Tumors (NET): Minimum Requirements for a NET Specialist. Cancer Treat. Rev..

[3] Rindi G., Mete O., Uccella S., Basturk O., La Rosa S., Brosens L.A.A., Ezzat S., de Herder W.W., Klimstra D.S., Papotti M. (2022). Overview of the 2022 WHO Classification of Neuroendocrine Neoplasms. Endocr. Pathol..

[4] Pobłocki J., Jasińska A., Syrenicz A., Andrysiak-Mamos E., Szczuko M. (2020). The Neuroendocrine Neoplasms of the Digestive Tract: Diagnosis, Treatment and Nutrition. Nutrients.

[5] Dasari A., Shen C., Halperin D., Zhao B., Zhou S., Xu Y., Shih T., Yao J.C. (2017). Trends in the Incidence, Prevalence, and Survival Outcomes in Patients with Neuroendocrine Tumors in the United States. JAMA Oncol..

[6] Modica R., Liccardi A., Minotta R., Cannavale G., Benevento E., Colao A. (2022). Therapeutic Strategies for Patients with Neuroendocrine Neoplasms: Current Perspectives. Expert Rev. Endocrinol. Metab..

[7] Clement D., Brown S., Leerdam M.V., Tesselaar M., Ramage J., Srirajaskanthan R. (2024). Sarcopenia and Neuroendocrine Neoplasms. Curr. Oncol. Rep..

[8] Altieri B., Di Dato C., Modica R., Bottiglieri F., Di Sarno A., Pittaway J.F.H., Martini C., Faggiano A., Colao A. (2020). Bone Metabolism and Vitamin D Implication in Gastroenteropancreatic Neuroendocrine Tumors. Nutrients.

[9] Laing E., Kiss N., Michael M., Krishnasamy M. (2020). Nutritional Complications and the Management of Patients with Gastroenteropancreatic Neuroendocrine Tumors. Neuroendocrinology.

[10] Kim T., Kim H. (2023). Pathophysiology and Therapeutic Management of Bone Loss in Patients with Critical Illness. Pharmaceuticals.

[11] Fiebrich H.B., Van Den Berg G., Kema I.P., Links T.P., Kleibeuker J.H., Van Beek A.P., Walenkamp A.M.E., Sluiter W.J., De Vries E.G.E. (2010). Deficiencies in Fat-Soluble Vitamins in Long-Term Users of Somatostatin Analogue. Aliment. Pharmacol. Ther..

[12] Massironi S., Zilli A., Bernasconi S., Fanetti I., Cavalcoli F., Ciafardini C., Felicetta I., Conte D. (2017). Impact of Vitamin D on the Clinical Outcome of Gastro-Entero-Pancreatic Neuroendocrine Neoplasms: Report on a Series from a Single Institute. Neuroendocrinology.

[13] Modica R., Benevento E., Altieri B., Minotta R., Liccardi A., Cannavale G., Di Iasi G., Colao A. (2024). Role of Bone Metastases in Lung Neuroendocrine Neoplasms: Clinical Presentation, Treatment and Impact on Prognosis. Int. J. Mol. Sci..

[14] Fazio N., Maisonneuve P., Frezza A.M., Ranallo N., Ibrahim T., La Salvia A., Brizzi M.P., De Divitiis C., Tafuto S., Pusceddu S. (2026). Bone Metastases from Neuroendocrine Neoplasms: Results of an Italian Nationwide Survey of Natural History and Management. J. Neuroendocrinol..

[15] Ghemigian A., Carsote M., Sandru F., Petca R.C., Oproiu A.M., Petca A., Valea A. (2021). Neuroendocrine Neoplasia and Bone (Review). Exp. Ther. Med..

[16] Liccardi A., Colao A., Modica R. (2025). Gender Differences in Lung Neuroendocrine Tumors: A Single-Center Experience. Neuroendocrinology.

[17] Zheng Z., Chen C., Jiang L., Zhou X., Dai X., Song Y., Li Y. (2019). Incidence and Risk Factors of Gastrointestinal Neuroendocrine Neoplasm Metastasis in Liver, Lung, Bone, and Brain: A Population-Based Study. Cancer Med..

[18] Lombard-Bohas C., Mitry E., O’Toole D., Louvet C., Pillon D., Cadiot G., Borson-Chazot F., Aparicio T., Ducreux M., Lecomte T. (2009). Thirteen-Month Registration of Patients with Gastroenteropancreatic Endocrine Tumours in France. Neuroendocrinology.

[19] Nuñez-Valdovinos B., Carmona-Bayonas A., Jimenez-Fonseca P., Capdevila J., Castaño-Pascual Á., Benavent M., Pi Barrio J.J., Teule A., Alonso V., Custodio A. (2018). Neuroendocrine Tumor Heterogeneity Adds Uncertainty to the World Health Organization 2010 Classification: Real-World Data from the Spanish Tumor Registry (R-GETNE). Oncologist.

[20] Scharf M., Petry V., Daniel H., Rinke A., Gress T.M. (2017). Bone Metastases in Patients with Neuroendocrine Neoplasm: Frequency and Clinical, Therapeutic, and Prognostic Relevance. Neuroendocrinology.

[21] Alexandraki K.I., Daskalakis K., Tsoli M., Grossman A.B., Kaltsas G.A. (2020). Endocrinological Toxicity Secondary to Treatment of Gastroenteropancreatic Neuroendocrine Neoplasms (GEP-NENs). Trends Endocrinol. Metab..

[22] Mazzilli R., Zamponi V., Mancini C., Giorgini B., Golisano B., Mikovic N., Pecora G., Russo F., Martiradonna M., Paravani P. (2025). Neuroendocrine Tumors and Diabetes Mellitus: Which Treatment and Which Effect. Endocrine.

[23] Hall L.A., Powell-Brett S., Thompson O., Smith D., Bradley E., Smith S., Vickrage S., Kemp-Blake J., Roberts K.J., Shah T. (2023). Casting a Wider NET: Pancreatic Exocrine Insufficiency Induced by Somatostatin Analogues among Patients with Neuroendocrine Tumours?. Cancers.

[24] Kailey B., van de Bunt M., Cheley S., Johnson P.R., MacDonald P.E., Gloyn A.L., Rorsman P., Braun M. (2012). SSTR2 Is the Functionally Dominant Somatostatin Receptor in Human Pancreatic β- and α-Cells. Am. J. Physiol. Endocrinol. Metab..

[25] Braun M. (2014). The Somatostatin Receptor in Human Pancreatic β-Cells. Vitam. Horm..

[26] Patel K.R., Nahar A., Elhassan Y.S., Shetty S., Smith S., Vickrage S., Kemp-Blake J., Palani R., Geh I., Venkataraman H. (2022). The Effects of Somatostatin Analogues on Glycaemia in the Treatment of Neuroendocrine Tumours. J. Neuroendocrinol..

[27] Natalicchio A., Faggiano A., Zatelli M.C., Argentiero A., D’Oronzo S., Marrano N., Beretta G.D., Acquati S., Adinolfi V., Di Bartolo P. (2022). Metabolic Disorders and Gastroenteropancreatic-Neuroendocrine Tumors (GEP-NETs): How Do They Influence Each Other? An Italian Association of Medical Oncology (AIOM)/Italian Association of Medical Diabetologists (AMD)/Italian Society of Endocrinology (SIE)/Italian Society of Pharmacology (SIF) Multidisciplinary Consensus Position Paper. Crit. Rev. Oncol. Hematol..

[28] Ni K., Yang J.Y., Baeg K., Leiter A.C., Mhango G., Gallagher E.J., Wisnivesky J.P., Kim M.K. (2021). Association between Somatostatin Analogues and Diabetes Mellitus in Gastroenteropancreatic Neuroendocrine Tumor Patients: A Surveillance, Epidemiology, and End Results-Medicare Analysis of 5235 Patients. Cancer Rep..

[29] Saif M.W., Romano A., Smith M.H., Patel R., Relias V. (2020). Chronic Use of Long-Acting Somatostatin Analogues (SSAs) and Exocrine Pancreatic Insufficiency (EPI) in Patients with Gastroenteropancreatic Neuroendocrine Tumors (GEP-NETs): An Under-Recognized Adverse Effect. Cancer Med. J..

[30] Lamarca A., McCallum L., Nuttall C., Barriuso J., Backen A., Frizziero M., Leon R., Mansoor W., McNamara M.G., Hubner R.A. (2018). Somatostatin Analogue-Induced Pancreatic Exocrine Insufficiency in Patients with Neuroendocrine Tumors: Results of a Prospective Observational Study. Expert Rev. Gastroenterol. Hepatol..

[31] Rinzivillo M., De Felice I., Magi L., Annibale B., Panzuto F. (2020). Occurrence of Exocrine Pancreatic Insufficiency in Patients with Advanced Neuroendocrine Tumors Treated with Somatostatin Analogs. Pancreatology.

[32] Cao Y., Dong B., Li Y., Liu Y., Shen L. (2025). Association of Type 2 Diabetes with Osteoporosis and Fracture Risk: A Systematic Review and Meta-Analysis. Medicine.

[33] Kamrul-Hasan A.B.M., Bhattacharya S., Ganakumar V., Nagendra L., Dutta D., Aalpona F.T.Z., Pappachan J.M. (2025). Trabecular Bone Score in Type 2 Diabetes Mellitus: An Updated Systematic Review and Meta-Analysis. J. Clin. Densitom..

[34] Pusceddu S., Vernieri C., Di Maio M., Prinzi N., Torchio M., Corti F., Coppa J., Buzzoni R., Di Bartolomeo M., Milione M. (2022). Impact of Diabetes and Metformin Use on Enteropancreatic Neuroendocrine Tumors: Post Hoc Analysis of the Clarinet Study. Cancers.

[35] Pusceddu S., Corti F., Prinzi N., Nichetti F., Ljevar S., Busico A., Cascella T., Leporati R., Oldani S., Pircher C.C. (2023). Safety and Antitumor Activity of Metformin plus Lanreotide in Patients with Advanced Gastro-Intestinal or Lung Neuroendocrine Tumors: The Phase Ib Trial MetNET2. J. Hematol. Oncol..

[36] Modica R., Liccardi A., Zamponi V., Gagliardi A., Nistor A., Veroi G., Arecco A., Faggiano A., Colao A. (2026). From Diabetes to Tumor Growth: Unravelling the Impact of Glucose-Lowering Therapies. Endocrine.

[37] Motylewska E., Gawronska J., Niedziela A., Melen-Mucha G., Lawnicka H., Komorowski J., Swietoslawski J., Stepien H. (2016). Somatostatin Analogs and Tumor Localization Do Not Influence Vitamin D Concentration in Patients with Neuroendocrine Tumors. Nutr. Cancer.

[38] Brunetti A., Cellini M., Lavezzi E., Zerbi A., Ferrillo G., Birtolo M.F., Berruti A., Cavati G., Lagana M., Gennari L. (2025). Fragility Fractures in Well-Differentiated Gastroenteropancreatic Neuroendocrine Tumors: Results from a Multicentered Retrospective Study. J. Neuroendocrinol..

[39] Aktypis C., Yavropoulou M.P., Efstathopoulos E., Polichroniadi D., Poulia K.A., Papatheodoridis G., Kaltsas G. (2025). Bone and Muscle Mass Characteristics in Patients with Gastroenteropancreatic Neuroendocrine Neoplasms. Endocrine.

[40] Marasco M., Dell’Unto E., Laviano A., Campana D., Panzuto F. (2024). Gastrointestinal Side Effects of Somatostatin Analogs in Neuroendocrine Tumors: A Focused Review. J. Gastroenterol. Hepatol..

[41] Clement D.S.V.M., van Leerdam M.E., de Jong S., Weickert M.O., Ramage J.K., Tesselaar M.E.T., Srirajaskanthan R. (2023). Prevalence of Sarcopenia and Impact on Survival in Patients with Metastatic Gastroenteropancreatic Neuroendocrine Tumours. Cancers.

[42] Casabella A., Hasballa I., Arecco A., Boschetti M., Della Sala L., Demontis D., Sulli A., Vera L., Veresani A., Ferone D. (2026). Bone Health in Neuroendocrine Tumors and Prognostic Implications beyond Skeletal Metastases. Front. Endocrinol..

[43] Putzer D., Gabriel M., Henninger B., Kendler D., Uprimny C., Dobrozemsky G., Decristoforo C., Bale R.J., Jaschke W., Virgolini I.J. (2009). Bone Metastases in Patients with Neuroendocrine Tumor: 68Ga- DOTA-Tyr3-Octreotide PET in Comparison to CT and Bone Scintigraphy. J. Nucl. Med..

[44] Strzelczyk J., Wójcik-Giertuga M., Strzelczyk J.K., Seńkowska A.P., Biernacki K., Kos-Kudła B. (2023). Selected Parameters of Bone Turnover in Neuroendocrine Tumors—A Potential Clinical Use?. J. Clin. Med..

[45] Gupta P.S., Grozinsky-Glasberg S., Drake W.M., Akker S.A., Perry L., Grossman A.B., Druce M.R. (2014). Are Serotonin Metabolite Levels Related to Bone Mineral Density in Patients with Neuroendocrine Tumours?. Clin. Endocrinol..

[46] Scopel M., De Carlo E., Bergamo F., Murgioni S., Carandina R., Cervino A.R., Burei M., Vianello F., Zagonel V., Fassan M. (2022). Bone Metastases from Neuroendocrine Tumors: Clinical and Biological Considerations. Endocr. Connect..

[47] Altieri B., Di Dato C., Martini C., Sciammarella C., Di Sarno A., Colao A., Faggiano A., Albertelli M., Ambrosetti E., Bianchi A. (2019). Bone Metastases in Neuroendocrine Neoplasms: From Pathogenesis to Clinical Management. Cancers.

